# Addressing Complex Symptom Burden in Aging and Long COVID Through a Remote Yoga and Self-Management

**DOI:** 10.1093/geroni/igaf122.4338

**Published:** 2025-12-31

**Authors:** Jennifer Portz, Christine Fruhauf, Stacey Schepens, Arlene Schmid

**Affiliations:** University of Colorado School of Medicine, Aurora, Colorado, United States; Colorado State University, Fort Collins, Colorado, United States; University of Southern California, Los Angeles, California, United States; Colorado State University, Fort Collins, Colorado, United States

## Abstract

Older adults with multiple chronic conditions (MCC) represent a population at particular risk for the long-term consequences of COVID-19. MCC are frequently accompanied by overlapping symptoms such as fatigue, pain, and depression, which long COVID can both mimic and intensify, accelerating functional decline and reducing overall quality of life. Older adults with MCC are not only more likely to develop long COVID but also experience greater difficulty recovering due to the complexity of managing multiple conditions simultaneously. Despite emerging guidelines, effective MCC/Long-COVID treatment strategies remain limited. This research evaluated the feasibility, usability, and preliminary impact of MY-Skills Mobile, a novel, 8-week, fully remote intervention that integrates therapeutic yoga with digital self-management education to improve physical, emotional, and social well-being across populations with high symptom burden. Two single-arm pilot studies tested MY-Skills Mobile: Study 1 enrolled 24 older adults (mean age 70±5) with MCC experiencing depressive symptoms, along with 9 care partners, and Study 2 involved patients (mean age 55.8±10.9) with long COVID (N = 9). Both studies met feasibility benchmarks for recruitment, retention, and attendance. Participants rated the intervention positively, noting benefits such as relaxation, increased energy, and emotional relief. Improvements were observed in depressive symptoms, fatigue, pain interference, and self-efficacy, with care partners reporting stronger social support. No adverse events occurred, though suicide risk screening identified five participants needing follow-up. MY-Skills Mobile represents an innovative, scalable model for delivering support to populations with complex, overlapping health concerns. Future research will evaluate efficacy through fully powered trials.

